# Dual inhibition of CDK4 and FYN leads to selective cell death in KRAS-mutant colorectal cancer

**DOI:** 10.1038/s41392-019-0088-z

**Published:** 2019-11-29

**Authors:** Yan Wang, Rongjie Lin, Huan Ling, Yuan Ke, Yangyang Zeng, Yudi Xiong, Qian Zhou, Fuxiang Zhou, Yunfeng Zhou

**Affiliations:** 1 Hubei Key Laboratory of Tumor Biological Behaviors, Wuhan, China; 2 Department of Radiation Oncology, Fujian Medical University Cancer Hospital, Fujian Cancer Hospital, Fuzhou, China; 3 Department of Radiation and Medical Oncology, Zhongnan Hospital of Wuhan University, Wuhan, China

**Keywords:** Gastrointestinal cancer, Cancer therapy

**Dear Editor,**

Activating mutations in KRAS have been found in nearly 50% of colorectal cancers (CRCs), and patients with KRAS mutations are resistant to the anti-EGFR agent cetuximab^1^. Currently, though there are several strategies targeting the mutated cysteine in KRAS (G12C; http://clinicaltrials.gov/ct2/show/NCT03600883), the frequency of the KRAS G12C mutation is much lower in CRC than in lung adenocarcinoma^2^. Thus, it is urgent to increase efforts to develop a novel strategy for KRAS-mutant CRC.

Protein kinases play a significant role during tumorigenesis and progression^3^. CDK4, in combination with RAS, generates malignant human epidermal tumorigenesis^4^. FYN mediates RAS activation (Jun-ichi Abe and Bradford C. Berk, 1999), and as a driver of epithelial−mesenchymal transition (EMT), FYN is strongly linked to the metastatic process for CRC^5–7^.

Considering the important roles of CDK4 and FYN in CRC, we first examined whether the dual inhibition of the kinases CDK4 and FYN using siRNAs led to more KRAS-mutant CRC cell death. SW620 (KRAS G12V), DLD-1 (KRAS G13D) and KRAS wild-type CRC (HCT8 and HT29) cells were transfected with CDK4 siRNA, FYN siRNA, both CDK4 siRNA and FYN siRNA, or scramble control siRNA for 48 h; then, immunoblotting was performed to verify if these siRNAs specifically reduced the expression of CDK4 and FYN (Fig. s1a). These cells were collected to evaluate cell death. Compared to the scrambled control siRNA, the dual depletion of CDK4 and FYN induced cellular apoptosis in KRAS-mutant CRC cells (*P* < 0.001) but not in KRAS wild-type CRC cells (*P* > 0.05) (Fig. 1a and s1b).

**Fig. 1:**
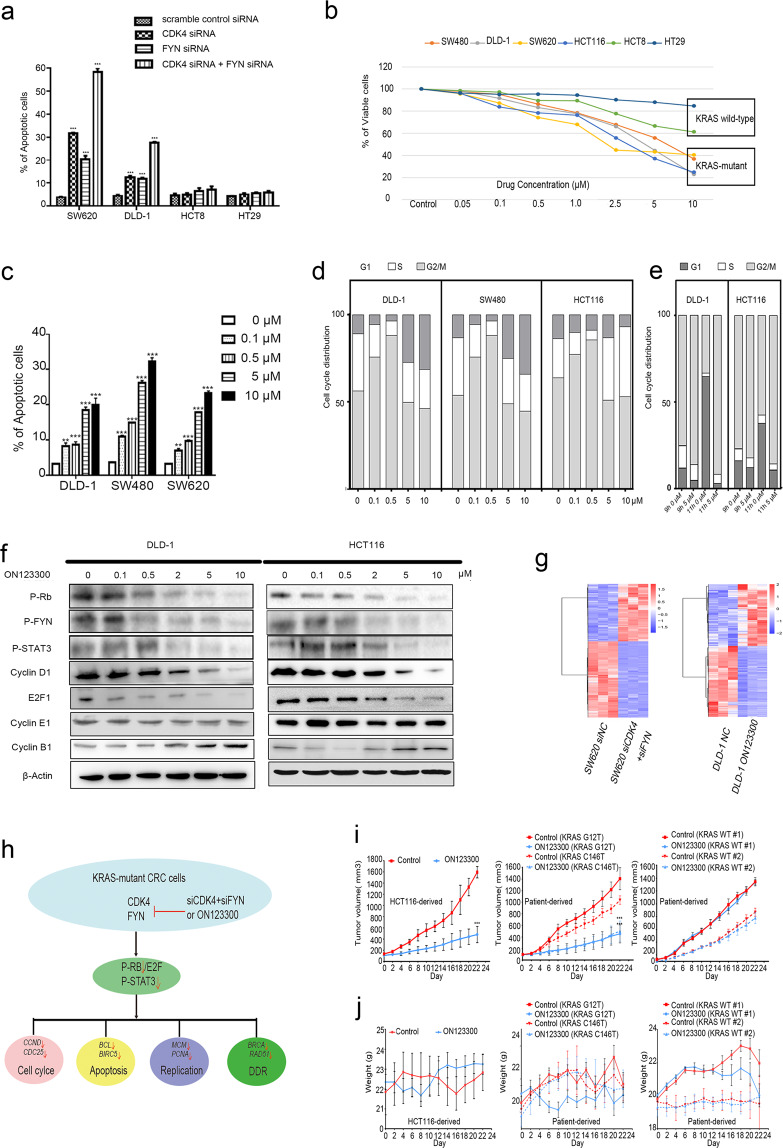
**a** Bar graphs show percent cells of apoptosis as determined in SW620, DLD-1, HCT8, and HT29 cells transfected with siRNA in (Fig. s1a)(SDs from three independent experiments). **b** Cell viability of SW480, DLD-1, SW620 and HCT116 cells (KRAS-mutant CRC), HCT8 and HT29 (KRAS wild-type CRC) treated with vehicle (DMSO) or increasing concentrations of ON123300 for 24 h (SDs from three independent experiments). **c** Bar graphs show percent cells of apoptosis as determined in Fig. s3A. (SDs from three independent experiments). **d** Cell cycle distribution of DLD-1, SW480, and HCT116 cells treated with increasing concentration of ON123300 for 24 h evaluated by flow cytometry (control: DMSO treated; SDs from three independent experiments). **e** Cell cycle distribution of synchronized DLD-1 and HCT116 cells incubated with vehicle (DMSO) or 5 μM ON123300 evaluated by flow cytometry (SDs from three independent experiments). **f** DLD-1 and HCT116 cells were treated with vehicle (DMSO) or an increasing concentration of ON123300 for 24 h, and the cell lysates were analyzed by western blotting using the indicated antibodies. β-Actin is shown as a loading control. **g** RNA sequencing data from dual inhibition CDK4 and FYN by siRNA identified 1653 differentially expressed genes (DEG) in SW620 cells, and ON123300-treated cells identified 1108 DEG in DLD-1 cells. **h** Schematic represents the mechanism of dual inhibition CDK4 and FYN in the control of KRAS-mutant CRC cell fate is shown. **i** The quantification of mean tumor volumes of control- and ON123300-treated KRAS-mutant and KRAS wild-type CRC mouse cohorts, *n* = 4 per group. **j** The body weight of the mice was measured during the treatment period every alternate day. **P ≤ 0.01, ***P ≤ 0.001.

Then, KRAS-mutant and KRAS wild-type CRC cells were treated with vehicle (DMSO) or ON123300, a dual CDK4/FYN inhibitor, for 24 h. ON123300 resulted in dramatically selective inhibition of KRAS-mutant CRC cell proliferation (Fig. 1b). The representative morphology of the cells is shown in Fig. s2a. The IC50 values of the cells are summarized in Table s1. As ON123300 is a multikinase inhibitor, we further verified that the antiproliferative effects are due to the inhibition of the kinase activity of FYN and CDK4. At 48 h after transfection with FYN siRNA or scramble control siRNA, SW620 and DLD-1 cells were treated with ON123300 for 24 h, and viable cells were counted. With increasing ON123300 concentration, the two cell growth curves nearly coincided (Fig. s2b).

KRAS-mutant cells were treated with DMSO or increasing concentrations of ON123300 for 24 h and then subjected to flow cytometry. We observed that all the KRAS-mutant CRC cell lines treated with ON123300 presented apoptosis (*P* < 0.01 or <0.001) (Fig. 1c and s3a). The levels of the apoptosis-resistant proteins Bcl-2 and survivin and the oncoprotein c-myc were decreased significantly in ON123300-treated SW480 and DLD-1 cells (Fig. s3b). All the KRAS-mutant CRC cells were obviously arrested in the G1 phase when they were treated with low concentrations of ON123300, and CRC cells accumulated significantly in the G2/M phase when treated with high concentrations of ON123300 (Fig. 1d and s4). Synchronized DLD-1 and HCT116 cells were used to further verify the results of the cell cycle analysis at high concentrations of ON123300. Compared to the control treatment at the same time point, ON123300 treatment resulted in a significant prolongation of the mitosis phase (Fig. 1e and s5). The results of immunoblot showed an obvious reduction in the levels of P-RB, cyclin D1, P-FYN and P-STAT3 and an increase in the levels of cyclin B1 at high concentrations of ON123300 in DLD-1 and HCT116 cells (Fig. 1f). We also observed significantly increased DNA damage in HCT116 cells treated with 5 µM ON123300 (51.145%; class nucleoids 3) compared to control cells (0.180%; class nucleoids 0) (proportion of DNA in the comet tail (TDNA %)) (Fig. s6a, b). Furthermore, we observed an obvious increase in the levels of the DNA damage marker γ-H2AX and a decrease in the levels of the DNA homologous recombination repair (HRR) hallmark proteins RAD51 and BRCA2 and the DNA replication proteins PCNA and MCM2 in ON123300-treated DLD-1 and HCT116 cells (Fig. s6c). Whole-transcriptome RNA-seq identified 1653 and 1108 differentially expressed genes (DEG) between the dual inhibition and control groups of SW620 and DLD-1 cells, respectively (Fig. 1g). Then, these DEGs were further annotated by KEGG pathway analysis (Fig. s7). The eight most significantly enriched pathways for these downregulated genes are shown in Tables s2 and s3. A schematic model depicting the mechanism of the dual inhibition of CDK4 and FYN in the control of KRAS-mutant CRC cell fate is shown in Fig. 1h.

Compared to control, ON123300 selectively inhibited the growth of tumors in both KRAS-mutant HCT116-derived and CRC chemoresistant PDTXs (Fig. 1i) without adverse side effects (Fig. s8b–d). Small residual KRAS-mutant tumors from the ON123300-treated mice were generally highly vascularized with infiltrating inflammatory cells, as shown by hematoxylin-eosin staining (Fig. s8a). ON123300 treatment did not affect the body weight of mice compared to that of the controls (Fig. 1j).

In conclusion, our findings demonstrated that the dual inhibition of CDK4 and FYN resulted in selective cell death of KRAS-mutant CRC cells and that ON123300, a dual CDK4/FYN inhibitor, exerted specific and potent anticancer activity against KRAS-mutant CRC both in vitro and in vivo and without adverse side effects. Our study provides preclinical evidence that simultaneously targeting CDK4 and FYN is a promising therapeutic strategy for patients carrying KRAS mutations.

## Supplementary information

supplementary material
