# Supplementary material for: Dual inhibition of CDK4 and FYN leads to selective cell death in KRAS-mutant colorectal cancer
Source: Signal Transduct Target Ther. 2019 Nov 29;4:52. doi: 10.1038/s41392-019-0088-z (PMC6882822; doi:10.1038/s41392-019-0088-z)
Supplement: Supplementary file 1 — supplementary material [file 41392_2019_88_MOESM1_ESM.docx]

Supplementary Materials for

Dual inhibition of CDK4 and FYN leads to selective cell death in KRAS-mutant colorectal cancer

Yan Wang1*, Rongjie Lin1,3*, Huan Ling1, Yuan Ke1, Yangyang Zeng, Yudi Xiong, Qian Zhou, Fuxiang Zhou1,2, Yunfeng Zhou1,2

Correspondence to: Yunfeng Zhou, e-mail: [yfzhouwhu@163.com](mailto:yfzhouwhu@163.com)

**Materials and Methods**

*1. Cell Lines*

KRAS-mutant CRC cell lines: HCT116 (KRAS G13D), DLD-1(KRAS G13D), SW480(KRAS G12V) and SW620 (KRAS G12V),and KRAS wild-type CRC cell lines:HCT8 and HT29 cells were cultured in Dulbecco’s Modified Eagle Medium (DMEM) or RPMI (Hyclone) supplemented with 10% FBS (Hyclone) and 100μg/ml penicillin-streptomycin at 37°C under humidified conditions and 5% CO2. All cell lines were verified by standardized short tandem repeat analysis and were negative for mycoplasma contamination.

*2. SiRNA*

siRNA was purchased from Santa Cruz Biotechnology: CDK4 siRNA (sc-29261), FYN siRNA (sc-29321), scramble control siRNA(sc-37007). Lipofectamine 2000 (11668500, invitrogen) was used for the siRNA transfections according to the manufacturer’s protocol.

*3. Drug and Antibodies*

ON123300 was purchased from Selleckchem. Antibodies against CDK4, FYN, Cyclin D1, Cyclin B1, PCNA, Bcl-2, P-STAT3 and MCM2 were purchased from abcam. Antibody specific for Survivin was purchased from ABclonal. Antibody against BRCA2 was purchased from sigma. Antibodies specific for P-RB, phosphoTyr416/420[Src/Fyn] and γ-H2AX were purchased from Cell Signaling Technology. Antibodies against RAD51 and c-Myc were purchased from SantaCruz. Antibodies specific for Cyclin E1 and β-Actin were purchased from Proteintech.

*4. CCK-8 assay*

Cells were seeded at appropriate number in wells of 96-well plates and cultured in 100 μl culture medium overnight, ON123300 was added to these medium at the indicated concentrations, 10 μl CCK-8 was added to each well 24 hrs posttreatment, and the plates were incubated at 37°C for 4 hrs. The absorbance of triplicate wells was then read at 450 nm using a 96-well plate reader.

*5. Flow cytometric analysis*

Cells for cell cycle analysis were filled in 70% ethanol overnight, and then treated with 100 ul RNase (50 μg/ml) for 20 min before addition of 400 μl propidium iodide (50 μg/ml) followed by flow cytometry analysis. Apoptosis was performed using the FITC-Annexin V Apoptosis Detection Kit (F-6012, us everbright) according to the manufacturer’s instructions.

*6. Western blot*

Cells were lysed in RIPA buffer (50 mmol/L Tris-HCl (pH, 8), 300 mmol/L NaCl, 10% NP-40, 1% sodium Deoxycholate), and 0.1% SDS and a protease inhibitor cocktail.Protein was separated by sodium dodecyl sulphate‐polyacrylamide gel electrophoresis (SDS-PAGE), transferred to polyvinylidene difluoride (PVDF) membranes. After blocking with 5% skim milk for 1 hour, proteins were incubated with the indicated antibody at 4 °C overnight. After washed with TBST, the membranes were incubated with the secondary antibody for 2 hrs, then the antibody binding was detected with an enhanced chemiluminescence (ECL) kit.

*7. Cell cycle synchronization*

Exponentially growing DLD-1 and HCT116 cells were synchronized in DMEM plus 2.5 mM thymidine (Sigma) for 18 hrs. After 18 hrs, thymidine was removed by washing with 1X PBS for three times and adding fresh media. After 9 hrs, thymidine was added for final concentration of 2.5 mM and incubated for another 15 hrs. The time is indicated as 0 hour. Then cells were released for 5 hrs to enter mitosis phase and were incubated with vehicle (DMSO) or 5 μM ON123300 for another 4 hrs or 6 hrs.

*8. Comet assay*

HCT116 were treated with ON123300 at 5 μM or vehicle (DMSO) for 24 hrs, then cells were collected and resuspended at 1×106-7 cells/ml in cold PBS. Comet gel board was prepared. Finally, the coverslip was removed, and the cells were lysed in alkaline lysis buffer (1 mM Na2EDTA, 0.3 M NaOH, pH13) for 20 min. The slides in the lysis buffer were electrophoresed in a high pH electrophoresis buffer at 25 V, 300 mA for 20 min. The slides were then washed in a neutralization buffer (0.39 M Tris, pH 7.5) for 15 min. The slides were stained with 30 μg/ml ethidium bromide for 20 min, then they were observed with a fluorescent microscope and analyzed using Comet Assay Software Pect (CASP 1.2.3 beta 1).

*9. RNA-Seq analysis*

2 μg total RNAs were used for stranded RNA sequencing library preparation using KCTM Stranded mRNA Library Prep Kit for Illumina® (Catalog NO. DR08402, Wuhan Seqhealth Co., Ltd. China) following the manufacturer’s instruction. PCR products corresponding to 200-500 bps were enriched, quantified and finally sequenced on Hiseq X 10 sequencer (Illumina). The data discussed in this article have been deposited in the sequence read archive of NCBI with study accession number PRJNA528195.They were mapped to the reference genome of Homo sapiens(Homo_sapiens.GRCh38;ftp://ftp.ensembl.org/pub/release87/fasta/homo_sapiens/dna/)using STRA software (version 2.5.3a) with default parameters. Reads mapped to the exon regions of each gene were counted by featureCounts (Subread-1.5.1; Bioconductor) and then RPKMs were calculated. Genes differentially expressed between groups were identified by using the edgeR package (version 3.12.1). A FDR corrected p-value cutoff of 0.05 and Fold-change cutoff of 2 were used to judge the statistical significance of differences in gene expressions. Both gene ontology (GO) analysis and Kyoto encyclopedia of genes and genomes (KEGG) enrichment analysis for differentially expressed genes were implemented by KOBAS software (version: 2.1.1) with a corrected P-value cutoff of 0.05 to judge statistically significant enrichment. Alternative splicing events were detected by using rMATS (version 3.2.5) with a FDR value cutoff of 0.05 and an absolute value of Δψ of 0.05.

*10. HCT116 cell-derived and colorectal patient-derived xenograft (PDX)*

Nude mice and NSG mice were handled in accordance with the Novartis Institutes for BioMedical Research (NIBR) Animal Care and Use Committee protocols and regulations. The animal experiments were approved by the Institutional Animal Care and Use Committee of Wuhan University and performed following Institutional Guidelines and Protocols. Freshly tumor tissue were obtained from patient diagnosed as CRC in Zhongnan Hosiptal with approval by the Institutional Review Boards of the hospital and the informed consents from patients. Four samples, two samples from chemo-resistant patients with KRAS-mutant (KRAS G12T and KRAS C146T) CRC, and the other two from ones with *KRAS* wild-type CRC, were used in these studies. A total of 1.0×106 HCT116 cells in phosphate-buffered saline (PBS) or primary CRC cell suspensions mixed with an equal volume of ECM (Matrigel) were injected subcutaneously into the dorsal flank of the mice. When the tumors grew to approximately 100mm3 in size at nearly 14 days after injection of cancer cells, mice were randomly divided into two groups (n=4 per group): ON123300 group, which received a dose of 100 mg/kg p.o. every alternate day and control group, which received saline every alternate day for three weeks. Tumor volumes and body weights were recorded every two days. Tumor volumes were calculated using the following equation: tumor volume (in mm³) = (L × W²)/2.

*11. Statistical analysis*

The difference of measurement data was compared with the Student’s two-tailed unpaired t-test and a P value of less than .05 was considered statistically significant.


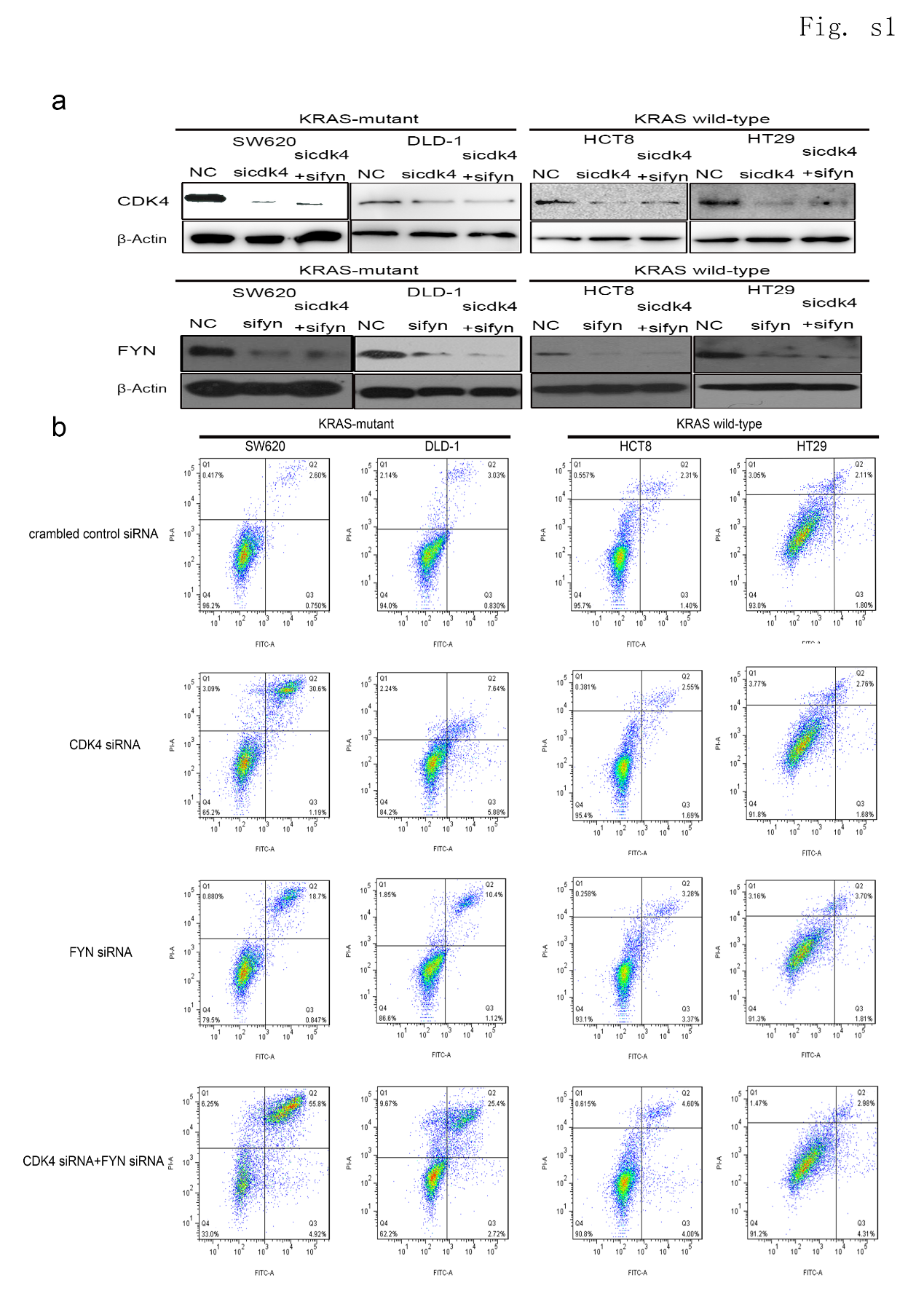


Fig. s1: (a) SW620, DLD-1, HCT8 and HT29 cells were transfected with CDK4 siRNA or (and) FYN siRNA or crambled control siRNA. After 48 hrs transfection, the cell lysates were analyzed by western blotting using the indicated antibodies. β-Actin is shown as a loading control. (b) Cell apoptosis of SW620, DLD-1, HCT8 and HT29 cells after transfected with siRNAs for 48 hrs evaluated by flow cytometry.


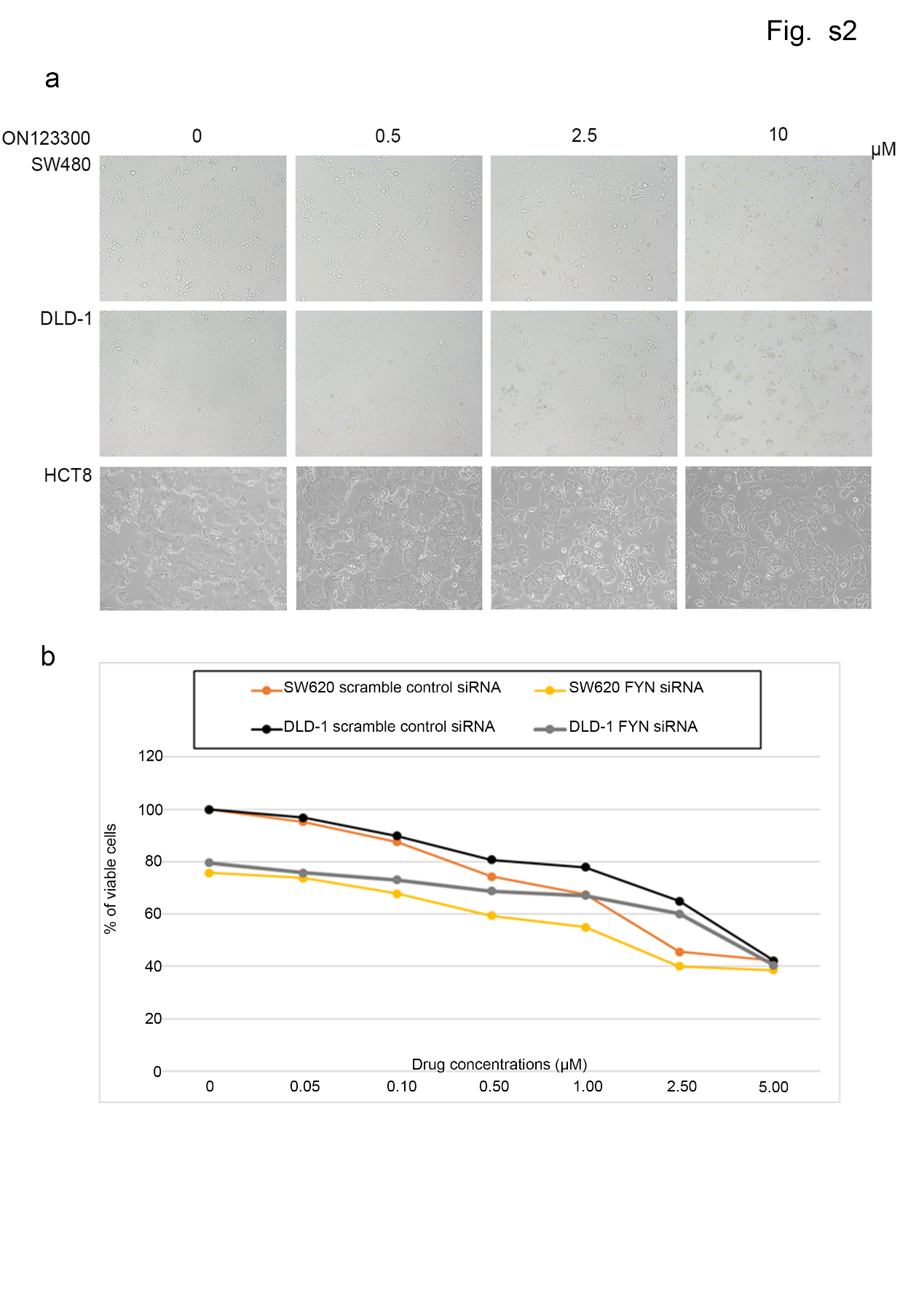


Fig. s2: (a) The morphology of SW480, DLD-1 and HCT8 cells treated with ON123300 (0.5, 2.5 and10 μM) or vehicle (DMSO) for 24 hrs. (b) Cell viability of SW620 and DLD-1 cells transfected with FYN siRNA or scramble control siRNA for 48 hrs and then were treated with incubated concentrations of ON123300 for 24 hrs.


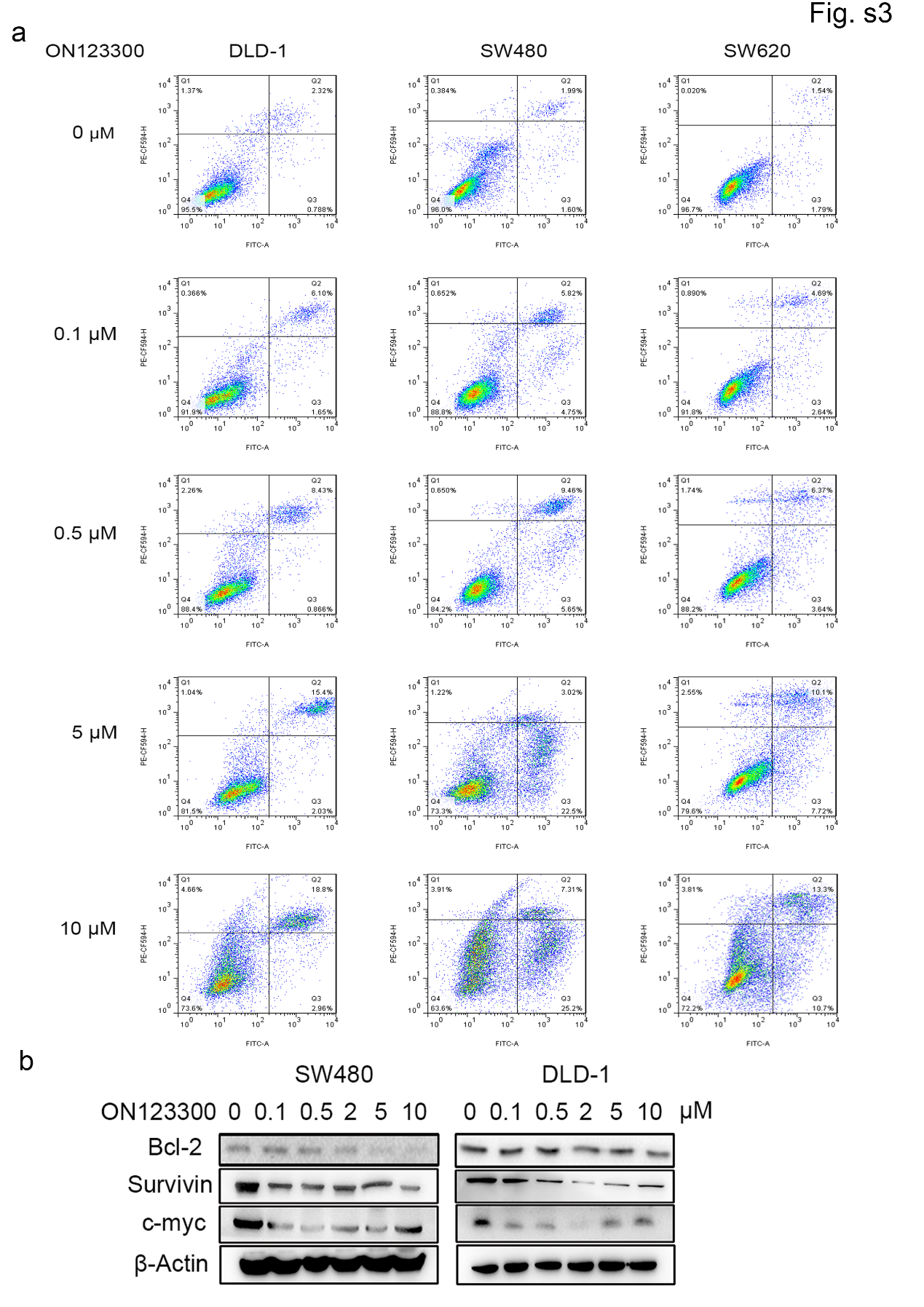


Fig. s3: (a) Cell apoptosis of SW480, DLD-1 and SW620 cells treated with increasing concentration of ON123300 for 24 hrs evaluated by flow cytometric analysis. (control: DMSO treated; SDs from three independent experiments).(b) SW480 and DLD-1 cells were treated with vehicle (DMSO) or increasing concentration of ON123300 for 24 hrs, western blot analysis was performed as described in Fig. s1a.


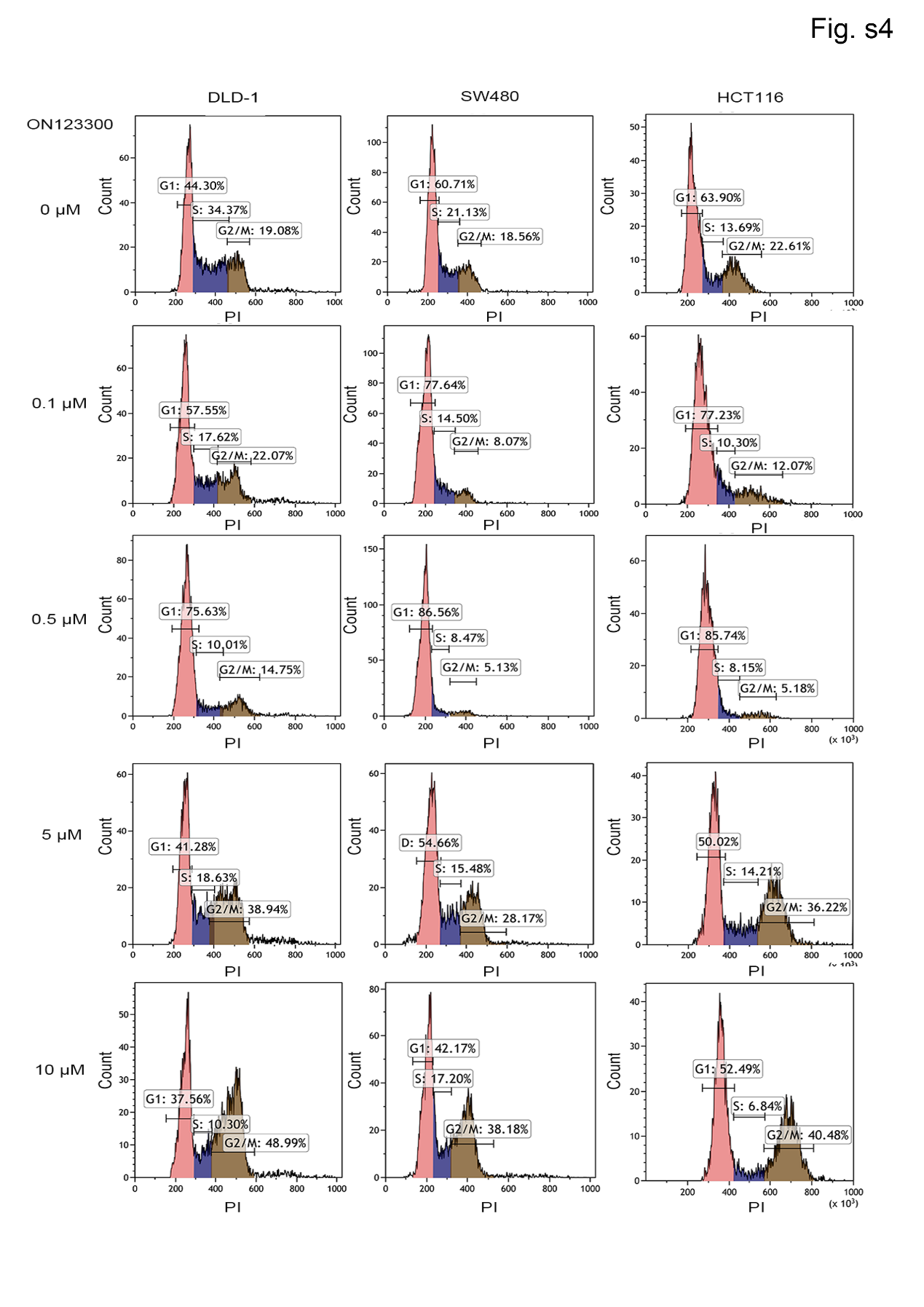


Fig. s4: Cell cycle of DLD-1, SW480 and HCT116 cells treated with vehicle (DMSO) or increasing concentration of ON123300 for 24 hrs evaluated by flow cytometry.


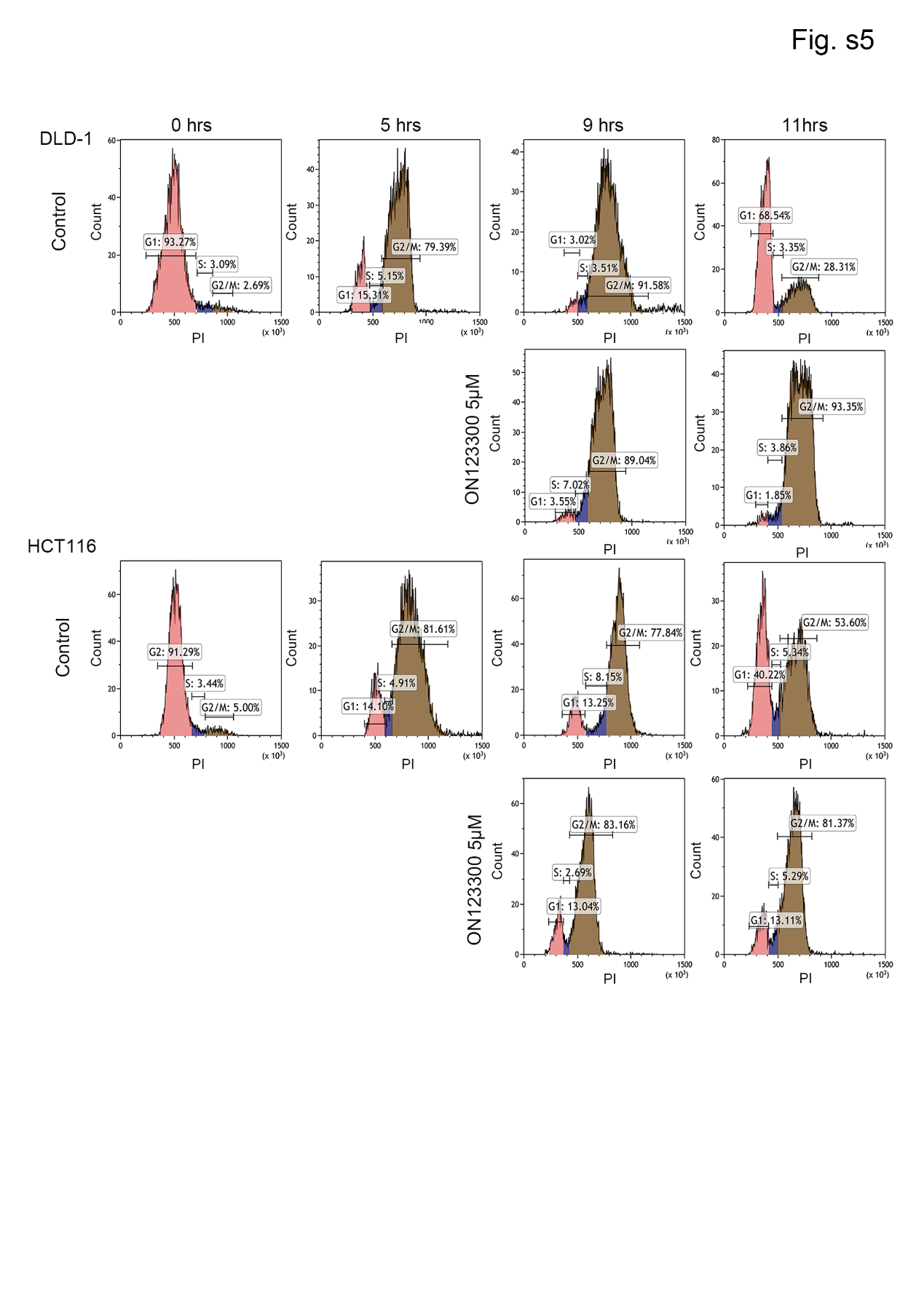


Fig. s5: Cell cycle of synchronized DLD-1 and HCT116 cells incubated with vehicle (DMSO) or 5 μM ON123300 evaluated by flow cytometry.


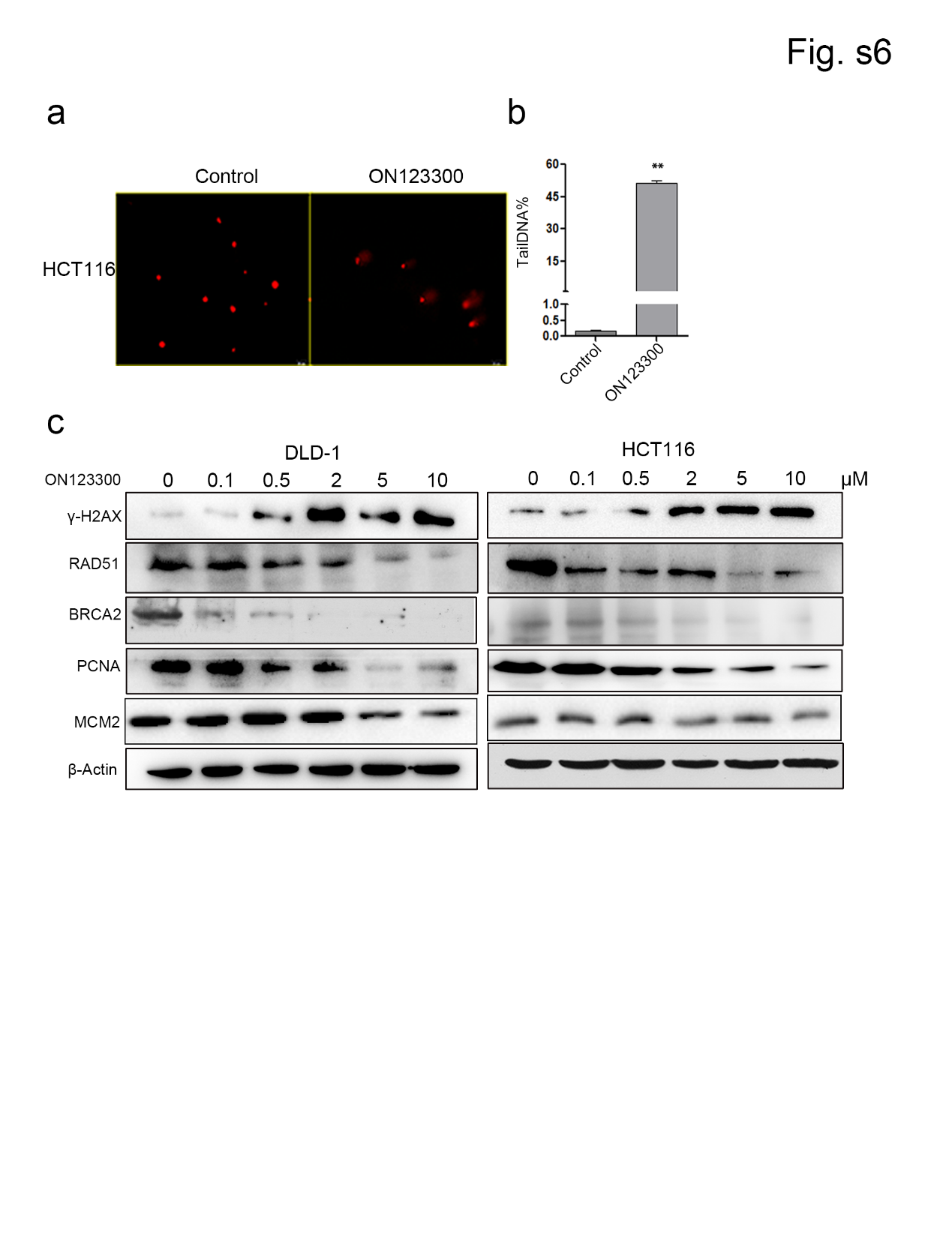
 Fig. s6: (a) HCT116 cells were treated with 5 μM of ON123300 for 24 hrs, DMSO-treated as control. Images of DNA damage was determined using Comet Assay kit for single-cell gel electrophoresis assay. Images representative of 3 independent experiments are shown. Magnifications, ×200; scale bar, 20 μm. (b) Proportions of DNA in the comet tail (TDNA%) in (a) **, P ≤ 0.01. (c) DLD-1 and HCT116 cell lines were treated with vehicle (DMSO) or increasing concentration of ON123300 for 24 hrs. Western blot analysis was performed as described in Fig. s1a.


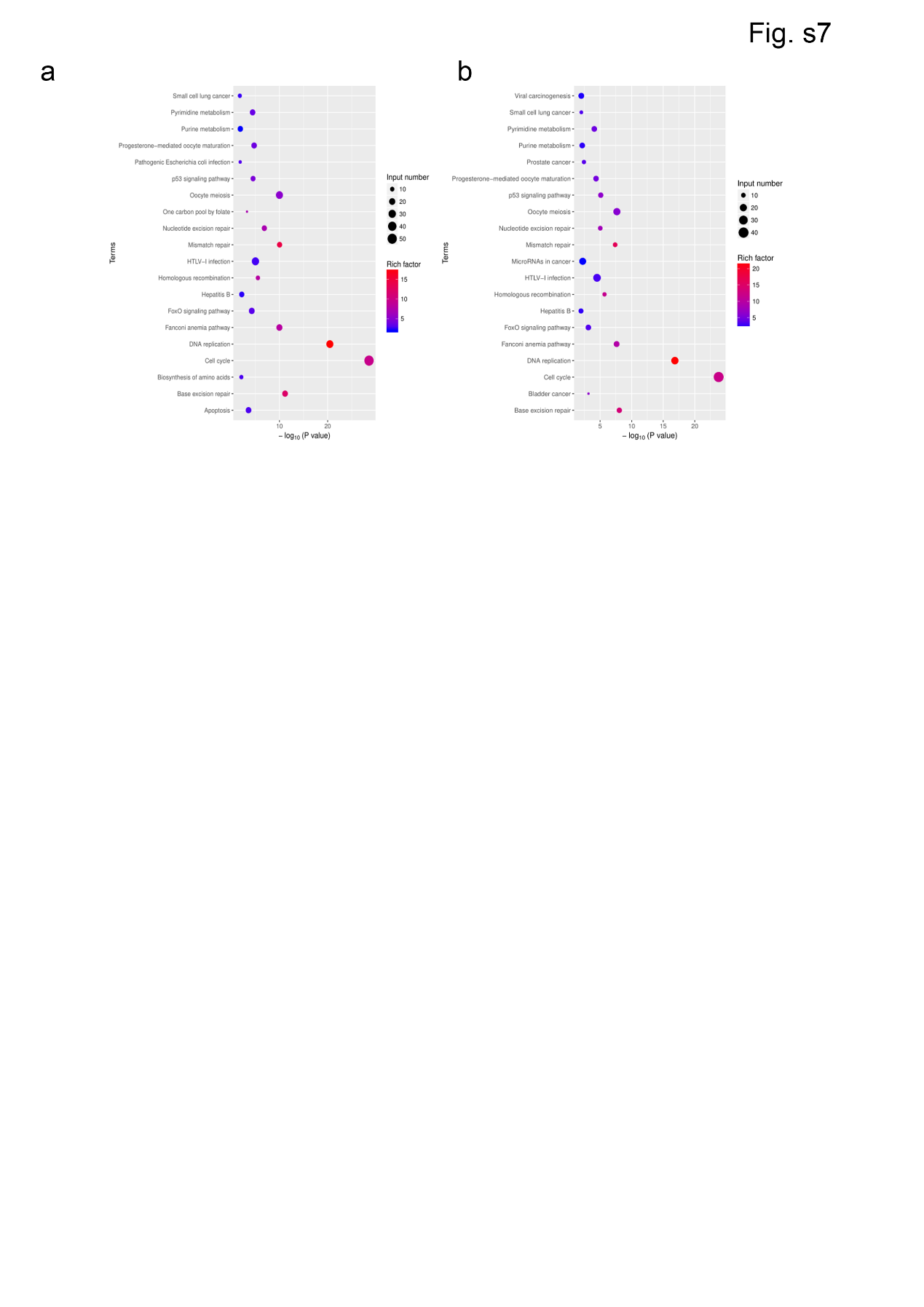


Fig. s7: (a) DEGs was further annotated by KEGG pathway analysis and enriched featured biologic pathways of down-regulated genes are shown in SW620 and (b) DLD-1 cells.


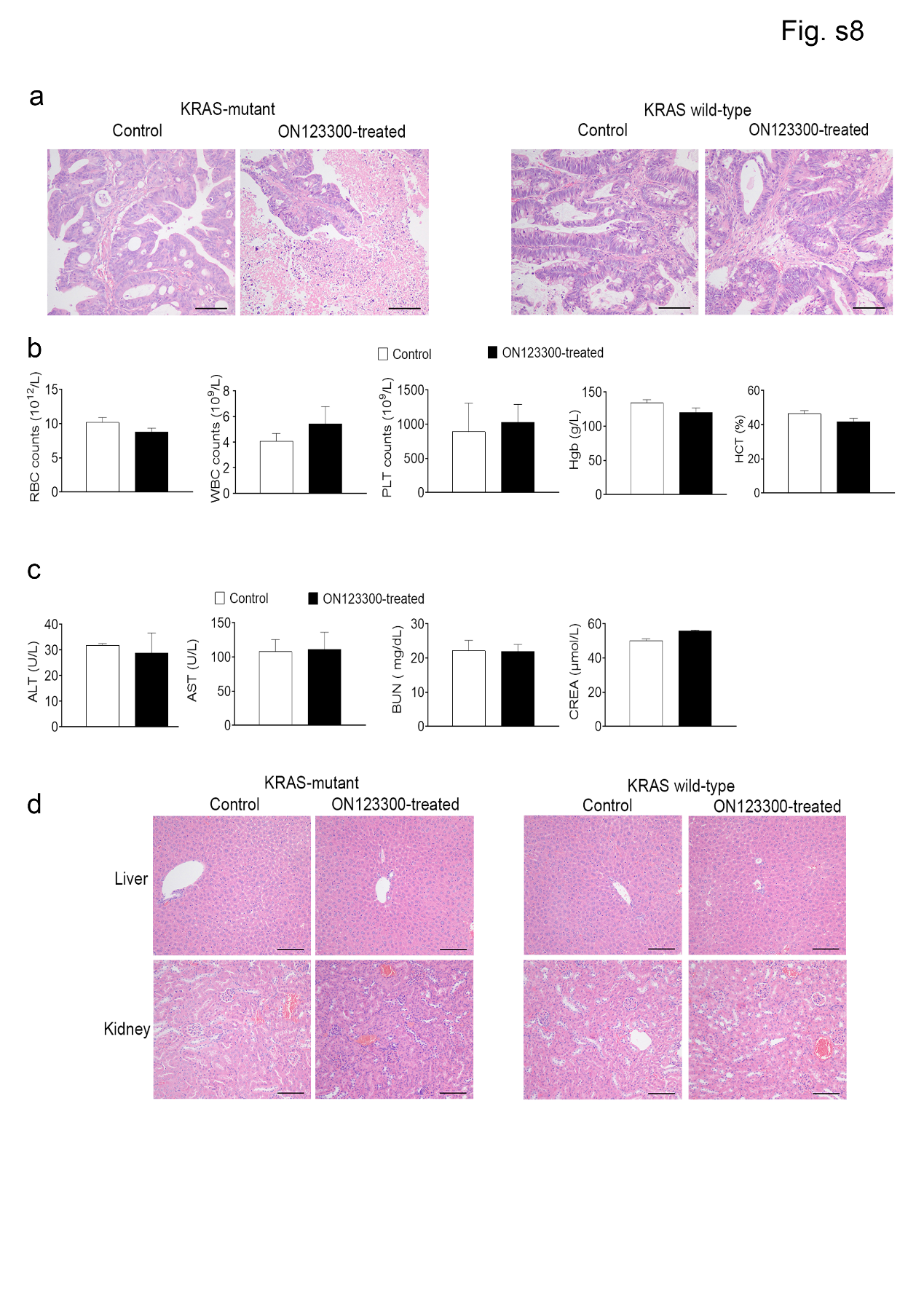
 Fig. s8: (a) The images of tumors with hematoxylin and eosin (control: DMSO). (b) The representative hematological assays in CRC xenograft mice treated with ON123300 compared to control. (c) The representative liver and renal function analysis and (d) liver and kidney histology in KRAS-mutant and KRAS wild-type CRC xenograft mice treated with ON123300 compared to control. Magnifications, ×200; scale bar, 100 μm.

**Table s1.** **The IC50 values of KRAS-mutant CRC cells.**

| **Cell lines** | **IC50（μM）**  **ON123300** |
| --- | --- |
| DLD-1 | 5.81 |
| HCT116 | 3.82 |
| SW480 | 3.61 |
| SW620 | 3.90 |

| **Table s2. The most significantly top 8 enriched pathways for down-regulated genes in SW620 cells.** | | |
| --- | --- | --- |
| **Term** | **P_Value** | **Corrected P_Value** |
| Cell cycle | 2.66*10^-29^ | 6.18*10^-27^ |
| DNA replication | 3.44*10^-21^ | 3.99*10^-27^ |
| Base excision repair | 6.81*10^-12^ | 5.27*10^-27^ |
| Mismatch repair | 9.37*10^-11^ | 3.96*10^-27^ |
| Oocyte meiosis | 9.78*10^-11^ | 3.96*10^-27^ |
| Fanconi anemia pathway | 1.03*10^-10^ | 3.96*10^-27^ |
| Nucleotide excision repair | 1.37*10^-07^ | 4.54*10^-27^ |
| Homologous recombination | 2.88*10^-06^ | 8.35*10^-27^ |
|  |  |  |
| **Table s3. The most significantly top 8 enriched pathways for down-regulated genes in DLD-1 cells.** | | |
| **Term** | **P_Value** | **Corrected P_Value** |
| Cell cycle | 1.64*10^-24^ | 3.40*10^-22^ |
| DNA replication | 1.39*10^-17^ | 1.44*10^-15^ |
| Base excision repair | 8.83*10^-09^ | 6.10*10^-07^ |
| Oocyte meiosis | 2.17*10^-08^ | 9.75*10^-07^ |
| Fanconi anemia pathway | 2.36*10^-08^ | 9.75*10^-07^ |
| Mismatch repair | 4.02*10^-08^ | 1.39*10^-06^ |
| Homologous recombination | 1.95*10^-06^ | 5.77*10^-05^ |
| p53 signaling pathway | 7.76*10^-06^ | 2.01*10^-04^ |
